# Spontaneous Pneumomediastinum on Bedside Ultrasound: Case Report and Review of the Literature

**DOI:** 10.5811/westjem.2015.1.24514

**Published:** 2015-03-13

**Authors:** Sybil Zachariah, Laleh Gharahbaghian, Phillips Perera, Nikita Joshi

**Affiliations:** Stanford University, Department of Emergency Medicine, Palo Alto, California

## Abstract

Spontaneous pneumomediastinum is a rare disease process with no clear etiology, although it is thought to be related to changes in intrathoracic pressure causing chest pain and dyspnea. We present a case of a 17-year-old male with acute chest pain evaluated initially by bedside ultrasound, which showed normal lung sliding but poor visualization of the parasternal and apical cardiac views due to significant air artifact, representing air in the thoracic cavity. The diagnosis was later verified by chest radiograph. We present a case report on ultrasound-diagnosed pneumomediastinum, and we review the diagnostic modalities to date.

## INTRODUCTION

Pneumomediastinum is defined as free air in the mediastinal cavity. It is considered spontaneous when there is no definite etiology, as opposed to secondary pneumomediastinum, which occurs with a clear causative factor. Spontaneous pneumomediastinum (SPM) is a rare condition with an incidence of less than 1:44,000.^1^ The pathogenesis of SPM is thought to involve a change in intrathoracic pressure that causes an alveolar leak that leads to a dissection of air along bronchovascular sheaths and eventually into the mediastinum.^2^ Patients often present with chest pain and dyspnea, which are nonspecific complaints and can be associated with an array of cardiopulmonary pathology presenting a challenge for the physician.

We report a case of SPM that was initially suspected by history and physical examination with supportive ultrasound findings, subsequently confirmed on chest radiograph. We then review the presentation, causes, and methods of diagnosis of pneumomediastinum, including a review of the literature of bedside ultrasound as a method of rapid evaluation.

## CASE REPORT

A 17-year-old male with no prior medical history presented to the emergency department (ED) with acute chest pain. The patient had been at work as a restaurant server when he noticed a sudden onset of substernal chest pain. The pain began while at rest without associated trauma, coughing, or sneezing. During initial examination in the ED, the patient was in significant pain and distress. He described the pain as pleuritic, substernal, positional, nonexertional, and worse with leaning forward. It was associated with shortness of breath. Additionally, he complained of a “crunching” sound in the right ear. He denied fevers, recent illness, cough, vomiting, leg swelling, drug use, alcohol use, and smoking.

The patient had a heart rate of 79 beats per minute, oral temperature of 36.9 degrees Celsius, respiratory rate of 16 breaths per minute, oxygen saturation of 99% on room air, and was initially noted to be hypertensive at 150/72mmHg. He was found to have a precordial crunching sound that was synchronous with the heartbeat audible on cardiovascular exam. His pulmonary exam was limited secondary to his inability to take deep breaths due to pain with inspiration. He had palpable crepitus on the right side of his neck. His initial electrocardiogram (EKG) showed small inferior and lateral q waves and was otherwise unremarkable. No prior EKG was available for comparison.

**Figure 1:**
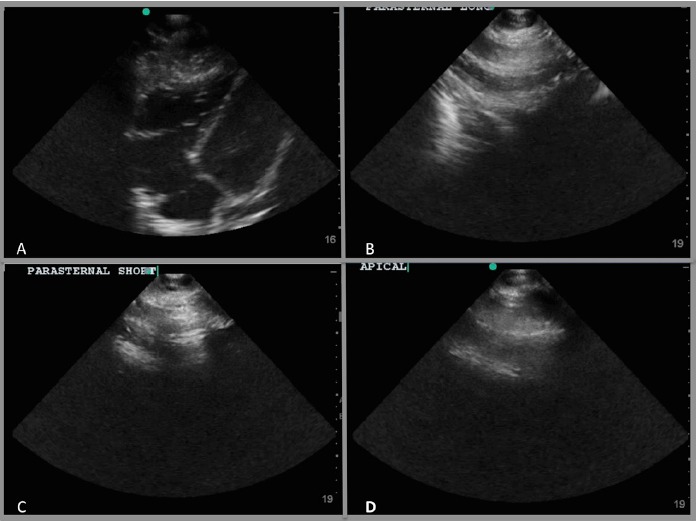
Bedside ultrasound images of patient with pneumomediastinum. (A) The subxiphoid view demonstrated normal cardiac contractility, normal chamber size, and lack of pericardial effusion. The parasternal long (B), parasternal short (C), and apical views of the heart were of poor quality with diffuse A lines (D), suggesting air artifact. These findings were suggestive of the presence of a pneumomediastinum.

**Figure 2:**
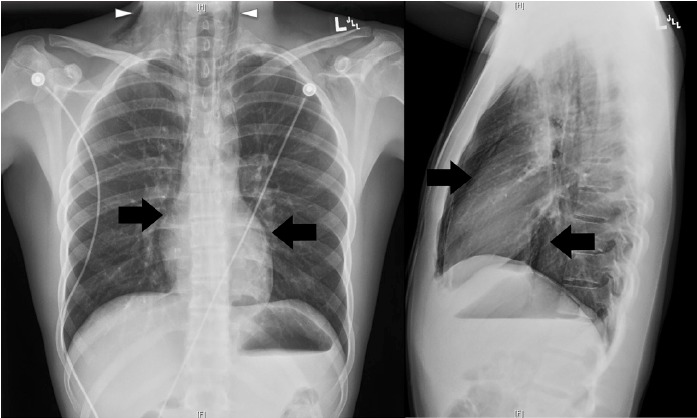
Chest radiograph, PA and lateral views. *PA*, posterior-anterior The patient’s pneumomediastinum was confirmed on chest x-ray, based on the presence of air dissecting along the mediastinum (arrows) and into the lower neck region bilaterally (arrow heads).

A bedside point-of-care ultrasound was performed (Figure 1, Video 1). The presence of bilateral lung sliding was confirmed with a linear transducer in the 2^nd^ intercostal spaces, thus decreasing the likelihood for a spontaneous pneumothorax. Using the phased array probe, the subxiphoid view demonstrated normal cardiac contractility, normal chamber size, and lack of pericardial effusion. However, parasternal long, parasternal short, and apical views of the heart were of poor quality with diffuse A lines, suggesting air artifact. These views were not visualized despite changes in patient positioning from supine to left lateral decubitus. At this point, concern was greatest for a pneumomediastinum with air dissecting anterior to the heart, causing poor sonographic windows. Pneumomediastinum was favored over pneumopericardium given the subxiphoid window remained clear and anatomy not obscured, suggesting the diaphragm, pericardium, and myocardium were still in contact and not separated by air. A chest radiograph was obtained and confirmed the diagnosis of pneumomediastinum, based upon the presence of air dissecting along the mediastinum and bilaterally into the neck and left axilla. (Figure 2) Given the patient’s findings, he was treated conservatively with oxygen by nasal cannula and admitted for observation with an unremarkable hospital course without requiring further intervention or imaging.

## DISCUSSION

The most common etiology of pneumomediastinum is trauma. Nontraumatic pneumomediastinum is spontaneous in approximately half of the cases in adult patients, more commonly in men.^3^ A recent retrospective chart review of pediatric patients with pneumomediastinum demonstrates that idiopathic primary pneumomediastinum is the most common etiology, seen most often in adolescent males.^4^ Younger children less than six years of age in this study were found to have secondary pneumomediastinum related to asthma, lower respiratory tract infection or pneumonia, foreign bodies, or croup.^4^

Chest pain is the most common presenting symptom in SPM, occurring in 72–75% of all patients with SPM, and up to 93% of pediatric patients. Other symptoms, such as dyspnea (48–59%) and cough (25–36%), were present less often. SPM has no known predisposing factors while secondary pneumomediastinum does, including smoking or tobacco use, asthma/chronic obstructive pulmonary disease (COPD), and interstitial lung disease or upper respiratory illnesses. Secondary pneumomediastinum can also be caused by increased intrathoracic pressure due to processes such as vomiting, strenuous activity, coughing, and childbirth.^2^–^4^

Subcutaneous emphysema or crepitus is a common finding noted on physical exam in up to 58% of patients. Hamman’s sign, or the crunching or bubbling sound over the mediastinum synchronous with the heart sounds, although nearly pathognomonic for pneumomediastinum and present in our patient, is present only in a minority of cases (18%). 2

Pneumomediastinum is most commonly diagnosed with chest radiographs (CXR), demonstrating free air tracking along the mediastinum or subcutaneous air in the shoulders or neck. Other radiological signs on CXR include the spinnaker sail sign or “angel wing sign,” more commonly seen in the pediatric population, due to the dissecting air elevating the thymus; the ring sign, due to air surrounding the pulmonary artery or its main branches; and the Naclieros V sign, due to a hyperlucent V shape between the descending aorta and the left hemidiaphragm.^2^,^5^

CXR reportedly misses or underestimates the severity of the SPM in 10–30% of cases.^6^–^8^ When CXR is equivocal but SPM is clinically suspected, computer tomography (CT) is generally considered the diagnostic standard to detect even small amounts of air in the mediastinum or subcutaneous tissues.^2^,^4^,^7^,^8^ In one retrospective review, lateral neck radiograph proved to be diagnostic in patients who had normal frontal CXRs, demonstrating pneumomediastinum in 9 out of those 10 cases. Overall, out of 21 neck radiographs performed, 20 detected subcutaneous emphysema or prevertebral air collection.^4^ However with national attempts to minimize radiation exposure, particularly in young patients, the utility of ultrasound has grown in many pulmonary applications; the identification of SPM by ultrasound is becoming increasingly common but is still in its early stages.^9^,^10^

Sonographic evaluation of pneumomediastinum was first described in 1983 as the “air gap sign,” described as a broad band of echoes during held respiration due to accumulating air obscuring normal cardiac structures, with drop out of echoes posteriorly. This sign was notable for appearing cyclically with the cardiac cycle.^11^ This sign was demonstrated in both pneumomediastinum and pneumopericardium. In 1994, a proposed mechanism to differentiate the two was described by the inability to see the heart with ultrasound in the subxiphoid view in pneumopericardium, as pericardial air extends inferiorly to the posterior reflection of the pericardium and scattering sound waves. However, in pneumomediastinum, the heart is well visualized in the subxiphoid view as it is in contact with diaphragm without obstructing air artifact.^12^ This was evident in our patient who had normal subxiphoid views but with difficult visualization of parasternal and apical views.

Other diagnoses to consider on the differential for air on the parasternal and apical windows include pneumothorax, severe COPD, pediatric causes of obstructive diseases such as alpha 1 anti-trypsin disease, or severe bullous diseases such as advanced tuberculosis. These processes will cause air-filled lung to move into position between the probe and the parasternal and apical cardiac windows. However, the subcostal view in these patients will be unaffected, as in this case of SPM.

In 1992, ultrasound was used to help delineate the diagnosis on a neonate with a CXR ambivalent for a medial pneumothorax and a pneumomediastinum. Ultrasound demonstrated a “fluorescent white” echogenic rim of air outlining the cardiac shadow on the left and the right at the subxiphoid view, along with a thin sliver of air outlining the left hemidiaphragm, thus confirming spontaneous pneumomediastinum.^13^

Other cases of SPM have been reported with results similar to ours, with parasternal and apical views not obtainable due to air artifact, with an associated normal subxiphoid view.^6^,^14^ SPM has been noted to be initially suspected due to ultrasound of the neck demonstrating both bullous hyperechoic artifacts around nerve sheaths extending along the aortic arch contour and “comet tails” or vertical gas artifacts in the anterolateral cervical region.^5^,^15^ Ultrasound has even identified a thin anechoic band identified as air, separating the pericardium from the pleural line.^5^

A recent study evaluated the use of ultrasound to evaluate for pneumomediastinum in neonates with abnormal mediastinal radiolucency on CXR. The study demonstrated not only the ability to detect the pneumomediastinum, but also identifies the location of air collection, which is most commonly the anterior margin of the thymus. This correlates with the raised thymus causing the spinnaker sail signs present on CXR.^16^

Management of SPM may include hospital admission for observation, especially for patients who are in distress, febrile, having worsening symptoms, or if secondary pneumomediastinum cannot be ruled out. Well-appearing patients with no concerning signs and symptoms may be treated with ambulatory care. Treatment generally consists of oxygen administration, pain control, and possible prophylactic antibiotics. More invasive intervention is rarely required and depends on the severity of symptoms or development of complications such as a tension pneumothorax.^8^,^17^

In conclusion, we have presented a case of spontaneous pneumomediastinum, which supports the use of bedside ultrasonography to aid in the diagnosis and rapid recognition of this less common cause of chest pain. When evaluating chest pain, SPM should be suspected when bedside echo demonstrates poor visualization of the heart with diffuse A lines in the parasternal and apical views in conjunction with normal visibility from the subxiphoid view. We can foresee in the future that ultrasound will be used more commonly to quickly evaluate for SPM in clinical practice, as well as more accurately diagnosed when evaluating for other thoracic disorders such as pneumothorax.

## Figures and Tables

**Video:** Spontaneous pneumomediastinum on bedside ultrasound.
